# Role of Satisfaction with Life, Sex and Body Mass Index in Physical Literacy of Spanish Children

**DOI:** 10.3390/children11020181

**Published:** 2024-02-01

**Authors:** Javier Urbano-Mairena, María Mendoza-Muñoz, Jorge Carlos-Vivas, Raquel Pastor-Cisneros, Antonio Castillo-Paredes, Miguel Rodal, Laura Muñoz-Bermejo

**Affiliations:** 1 Promoting a Healthy Society Research Group (PHeSO), Faculty of Sport Sciences, University of Extremadura, 10003 Caceres, Spain; jurbmai@unex.es (J.U.-M.); raquelpc@unex.es (R.P.-C.); 2 Research Group on Physical and Health Literacy and Health-Related Quality of Life (PHYQOL), Faculty of Sport Sciences, University of Extremadura, 10003 Caceres, Spain; 3 Physical Activity for Education, Performance and Health (PAEPH) Research Group, Faculty of Sport Sciences, University of Extremadura, 10003 Cáceres, Spain; jorgecv@unex.es; 4 Grupo Investigación en Actividad Física y Salud Escolar, Escuela de Pedagogía en Educación Física (AFySE), Facultad de Educación, Universidad de Las Américas, Santiago 8370040, Chile; antonio.castillo@udla.cl; 5 BioErgon Research Group, Faculty of Sport Sciences, University of Extremadura, 10003 Caceres, Spain; mrodal@unex.es; 6 Social Impact and Innovation in Health (InHEALTH), University Centre of Mérida, University of Extremadura, 06800 Merida, Spain; lauramunoz@unex.es

**Keywords:** children, physical fitness, physical activity, health, life satisfaction

## Abstract

Physical activity (PL) is essential to achieve good health, prevent cardiovascular diseases, obesity and overweight, as well as to achieve a better quality of life. Therefore, PL could become the tool to increase the practice of physical activity among young people, thus increasing life satisfaction (LS) given its positive relationship with physical activity. A single-measure cross-sectional correlational study was carried out, involving 135 children aged 8–12 years from Extremadura. They were administered the SWLS questionnaire and the Canadian assessment of physical literacy (CAPL-2). Significantly higher levels of PL (*p* = 0.010) were found in normal-weight children compared to overweight and obese children, due to the physical competence domain score (*p* < 0.001). PL was directly related to SWLS (*p* < 0.001), but inversely related to BMI (*p* = 0.018). The daily physical activity behaviour domain was explained by SWLS (*p* < 0.001) and sex (*p* < 0.001). Physical competence was described by SWLS (*p* < 0.001) and BMI (*p* = 0.045). Finally, the motivation and confidence domain were only significantly associated with SWLS (*p* < 0.001). It was concluded that boys and girls of normal weight achieved higher levels of PL and LS than those with overweight and obesity, establishing a negative relationship of PL with BMI and positive with LS.

## 1. Introduction

Nowadays, leading a sedentary lifestyle has become one of the major health risk factors [1], being one of the main causes of death in developed countries [2]. Numerous studies show that people who are more physically active have a lower risk of developing any type of disease and even a lower risk of death [3]. However, the level of physical activity among young people has decreased notably due to various reasons, including the emergence of electronic devices as a form of entertainment [4,5], reduced availability of active recreational activities [6], the use of non-active means of transport [4,5], and an environment that is often not conducive to the practice of physical activity [7,8].

Cardiovascular diseases, hypertension, type II diabetes [9,10,11], and mental health issues [12], such as depressive and/or anxiety disorders [13,14], are some of the diseases related to sedentary and inactive lifestyles. Similarly, overweight and obesity are also highly correlated [15,16]. Developed countries have the highest rates of childhood obesity [17], and the proportion of childhood obesity has increased dramatically [17]. This fact has led to sedentary lifestyles being studied as a determinant factor in childhood obesity [18].

In spite of all this, the practice of physical activity is fundamental for the preservation of good health [19,20], and all its benefits have been reported on many occasions, such as the improvement of cardiorespiratory capacity and muscular strength [11] and, above all, the improvement of quality of life [21] and well-being [22]. In addition, physical inactivity, together with the risk of obesity and overweight, are factors that are associated with poorer quality of life and lower life expectancy [23].

All this shows the need to establish strategies that promote active lifestyles among young people, with the need to reduce levels of sedentary lifestyles from childhood and adolescence [24,25], and with it, associated diseases such as obesity, overweight, and mental health care among the young population.

Several studies show that those who are physically active have fewer mental health disorders, in addition to achieving greater cognitive development [12,26]. Physical activity is considered an effective strategy for achieving a high quality of life [27] and physical activity not only enables young people and adolescents to achieve a high level of physical and mental health, but also to be satisfied with their lives.

Life satisfaction can be defined as the cognitive appraisal of an individual’s quality of life based on his or her own set of criteria [28] and can play a crucial role in the development of children and adolescents, as it has several benefits for their psychological well-being [29]. Some benefits reported by different studies in terms of high life satisfaction include motivating young people to explore their environment and contribute constructively to their development [30], more positive attitudes towards educators and the educational institution [31], greater mental engagement [32], and higher academic ambitions [33]. 

In addition to these benefits, several studies have established a strong relationship between physical activity and life satisfaction [34,35,36], showing that schoolchildren and adolescents who engaged in more hours of physical activity tended to feel more satisfied with their lives [37,38].

Therefore, physical literacy (PL) could become an ideal tool for children and adolescents to acquire the habit of long-term physical activity [39]. Although there are numerous definitions of PL [39], it could be defined as a person’s understanding, knowledge, physical competence, motivation, and ability to be physically active on a sustained basis [40,41], with PL being a tool to help understand why young people are or are not physically active [41] and to take action based on these findings.

The relationship between PL and sustained lifelong physical activity is still being explored, but there is no disagreement about its benefits, thanks to all the definitions and evaluations of PL that have been carried out [39]. Thus, several studies have shown that higher levels of PL are associated with improvements in body composition [42], blood pressure, and quality of life [43].

However, no studies in children have investigated the relationship between PL and life satisfaction (LF), despite its small association with physical activity. A previous study [44] shows a positive association between PL and important aspects of emotional and social well-being in adolescents, although it does not encompass LS. On the other hand, the latest results of the Health Behaviour in School-aged Children (HBSC) study [45] show that LS has decreased in recent years in this population, especially among girls. In this sense, it could be interesting to know the factors related to LS in order to identify which elements could expose girls to a higher risk of experiencing a low sense of satisfaction [46].

It should be noted that PA and PL are important factors that could influence the promotion of LS [47], and have been directly related to mental health and resilience (Ma, et al. [48]). In this regard, since it has been shown that the females report worse mental health [49] and self-esteem [50], poorer physical fitness [51] and less healthy BMI [51], it is considered necessary to analyse the influence of gender and fitness on the different domains of PL, as well as on weight categories and possible differences.

Therefore, the main objectives were (1) to explore PL and LS in children aged 8–12 years; (2) to analyse the differences between sexes and bodyweight groups; and (3) to examine the influence of sex, BMI and LS on PL and their respective domains. We hypothesised that significant differences between sexes and bodyweight state will be found in PL and LS. Moreover, PL will be positively correlated with LS and negatively correlated with BMI.

## 2. Materials and Methods

### 2.1. Design

A single-measure cross-sectional correlational study was conducted.

### 2.2. Participants

Ten schools from the autonomous community of Extremadura were contacted, and two of them decided to participate. The study aims and procedures were explained to the head of the centre, and they themselves contacted the parents or guardians of the pupils to obtain authorisation from those who decided to participate. Once authorisation and acceptance were obtained from the participants, two researchers went to the schools to carry out the evaluations. 

To be included into the study, schoolchildren needed to meet the following eligibility criteria: (1) age between 8 and 12 years; (2) consent of parents and/or guardians; (3) residence in Extremadura; and (4) no pathologies preventing the practice of physical activity. Thus, the final sample consisted of a total of 135 participants, of whom 53.4% were female and 46.6% were male, with a mean age of 10.09 (±0.76) years. 

### 2.3. Ethics

The research received the approval of the Bioethics and Biosafety Committee at the University of Extremadura (approval number: 138/2019, in accordance with the revisions made to the Helsinki Declaration by the 64th General Assembly of the World Medical Association (Fortaleza, Brazil, 2013) and in compliance with Law 14/2007 on Biomedical Research.

### 2.4. Procedures and Measures

The procedures were carried out following the Well-Being, Obesity and Motricity Observatory (WOMO) study protocol [52]. In addition, for the assessment of PL, the Canadian Assessment of Physical Literacy 2 (CAPL-2) assessment guide developed by the Healthy Active Living and Obesity Research Group (HALO), belonging to the Children’s Hospital of Eastern Ontario Research Institute [53], was followed in its adaption to Spanish [54]. The participants practised the physical test prior to the evaluation.

#### 2.4.1. Anthropometry

Standardised conditions were established for the measurements, according to those established by the WHO [55], and previously by the ALADINO study [56] and WHO European Childhood Obesity Surveillance Initiative (COSI) [57] reports. For both weighing and sizing, participants removed their shoes and socks, as well as any personal accessories (earrings, pendants, etc.). 

Body weight was measured using a bioimpedance meter (Tanita MC-780 MA, Tanita Corporation, Tokyo, Japan). For this purpose, the sex, age, and height of the participants were entered. Body weight was recorded in KG.

Height was obtained using a height measuring device (Tanita Tantois, Tanita Corporation, Tokyo, Japan). It was placed on a vertical surface perpendicular to the ground, recording the measurement in centimetres, and its approximation in millimetres. For this purpose, the participants stood upright, with their arms relaxed and the feet balanced. 

#### 2.4.2. Physical Literacy

The Canadian Assessment of Physical Literacy Development (CAPL-2) was applied to assess PL [58,59]. This assessment is composed of 4 different domains with a final score ranging from 0 to 100 points. These domains are physical competence, daily physical activity behaviour, knowledge and motivation, and confidence. Each domain is composed of different tests, making up a final score for each domain.

1. Physical competence. This domain assesses the physical ability of the participants through 3 different tests. Each test is evaluated with a score from 1 to 10, obtaining a final score for the domain out of 30 points. These tests are the Canadian Agility and Movement Skill Assessment (CAMSA) as an agility circuit; the abdominal plank isometric test [60] for 2 min; and the PACER (Progressive aerobic Cardiovascular Endurance Run) cardio-respiratory capacity test.
2. Daily physical activity behaviour. The total score for this domain is obtained by the number of total steps taken by participants recorded on an activity wristband (Xiaomi mi Band 3, Xiaomi Corporation, Beijing, China) and the number of minutes of physical activity undertaken by participants for at least 60 min.
3. Knowledge and understanding. The score for this domain is obtained by answering 5 questions, which can be scored between 1 and 10 points. Four of the five questions are multiple choice questions, scoring 0 to 1 point, and a final question which consists of filling in the blanks by completing a story, scoring 1 to 6 points.
4. Motivation and confidence. Domain composed of 4 parts: intrinsic motivation, predilection, suitability, and competence. This domain assesses participants’ confidence and motivation to be physically active, scoring from 1 to 30 points.

The domains of motivation and confidence and knowledge and understanding were assessed on the basis of the Spanish version of the questionnaire (Supplementary Materials), which showed high internal consistency (α = 0.730 to 0.970) and test–retest reliabilities ranging from moderate to almost perfect in the knowledge and understanding domain [61]. 

After the final score had been obtained, participants could be classified into 4 different levels of PL, according to the total score, gender, and age: (1) beginner; (2) progressing; (3) sufficient; (4) excellent. Beginner and progressing participants included schoolchildren without an adequate level of PL. Those with a successful score included schoolchildren who had reached the minimum level of PL, while children with an excellent level demonstrated a high level of PL [59].

#### 2.4.3. Life Satisfaction

The satisfaction with life survey (SWLS) [62] represents a multi-item assessment instrument used to quantify overall self-perceived LS. This measure is composed of five items, rated on a Likert scale ranging from 1 (strongly disagree) to 6 (strongly agree). The study subjects completed the Spanish version of the SWLS [63].

## 3. Results

Table 1 displays the characteristics of the sample and its comparison based on participants’ sex and bodyweight state. On the one hand, between-sex comparisons revealed significant greater score of males compared to females in the PL level (*p* < 0.001) and DB (*p* < 0.001), PC (*p* = 0.012) and MC (*p* = 0.020) domains. However, no differences were observed in the KU domain (*p* = 0.853) between sexes. Similarly, the total score in SWLS and the scores for all of its items were higher in males than in females (*p*: < 0.001 to 0.009), except for the item “The things in my life are excellent” (*p* = 0.128), where no differences were observed. Moreover, non-significant differences were observed in age and BMI (*p* < 0.05).

On the other hand, the comparison between bodyweight states showed a significant greater PL level (*p* = 0.010) among normal-weight children compared to overweight or obese children, mainly due to the superior score in the PC domain (*p* < 0.001), since there were non-significant differences in the rest of the PL domains (*p* > 0.05). Moreover, normal-weight children present a meaningful greater satisfaction with life than overweight or obese children. In fact, normal-weight individuals showed a superior score in all SWLS items (*p* = 0.023 to 0.043) than their overweight or obese counterparts, except for the item “I am happy with my life” (*p* = 0.745), where a similar score was observed.

Table 2 shows the stepwise regression modelling outcomes obtained for four different models for the total PL and its domains DB, PC and MC, respectively. The first model shows that PL can be explained by SWLS, sex, and BMI. Concretely, PL is directly associated with SWLS (β = 0.412; *p* < 0.001), but inversely related to sex (β = −0.301; *p* < 0.001) and BMI (β = −0.176; *p* = 0.018). The second model indicates that the DB domain is defined by SWLS and sex. Specifically, the DB domain is positively and significantly linked with SWLS (β = 0.299; *p* < 0.001), but inversely associated with sex (β = −0.358; *p* < 0.001). Likewise, the third model explains that the PC domain is associated with SWLS and BMI. The PC domain is only explained by SWLS (β = 0.377; *p* < 0.001), but is negatively linked to BMI (β = −0.166; *p* = 0.045). Finally, the MC domain is only explained by SWLS, and they are positively and significantly associated (β = 5.975; *p* < 0.001). Therefore, the SWLS is the only mediator variable common to PL and its domains.

## 4. Discussion

This study investigated sex and weight group differences in PL and LS in children and explored the effect of sex, BMI, and LS on PL and its domains. The main finding was a negative association between PL and BMI and sex, while for LS, the association was positive.

The majority of studies that evaluated PL differences between genders found that males scored higher than females on the CAPL-2; however, these studies did not find statistically significant differences between the two groups [64,65,66], which contrasts with our study’s results, which showed significant differences between the genders. Longmuir et al. [67] observed a significant interaction between age and gender for the CAPL total score, showing similarity between sexes at 10 years of age and earlier. While girls maintained the same mean total score at 11 years of age, boys’ mean score increased, and even at 12 years of age, the mean score continued to increase. This could be related to the age of the sample. In light of the fact that the mean age of the study participants was 10.09 years with a standard deviation of 0.76 years, it could be considered that the participants are at the age limit where such gender differences are most likely (10 years and older) [67]. In this line, when moving on to adolescence, these significant differences between genders were confirmed for almost all CAPL components [68]. Other similar studies found gender differences in some PL scores [43,69]. In terms of domain-specific scores, boys outperformed girls, which is consistent with the scientific literature that generally suggests that boys are more physically fit, motivated, and engaged in daily activities than girls. Li et al. [64] speculated that this disparity may result from boys engaging in more overall physical activity than girls [70].

The impact of BMI on PL and its domains has been studied scientifically on several occasions in various populations. Higher PL scores have been linked to improved body composition in children [43]. In this regard, Mendoza-Muñoz et al. [42] investigated the impact of BMI on PL in children aged 8–12 years, finding that overweight children had lower levels of PL than those who were not overweight, which is consistent with the findings of this study, where BMI was found to be inversely related to PL (*p* = 0.018). In the same vein, Delisle et al. [71] found that non-overweight children had significantly higher PL scores than overweight children, which is consistent with the findings of this study because when weight groups were separated, normal-weight children had significantly higher PL scores than overweight children (*p* = 0.010). Nonetheless, while non-overweight children scored higher than overweight children across the board, substantially higher scores were found only in the physical competence domain (*p* < 0.001). Numerous studies have found that non-overweight children had superior physical fitness than overweight children [72,73]. This might be attributed, as MacDonald et al. [74], suggested, to the fact that persons with higher scores are more likely to participate in more sporting activities than those with lower scores, and hence have superior cardiorespiratory fitness gained from more physical activity. Furthermore, physical skill is the most important factor related to fitness level [75].

Numerous studies on LS in children have been conducted, but the results have been conflicting when it comes to gender differences [76]. In this context, Breslin et al. [77] discovered that boys reported higher LS than girls in their research with children aged 9–11 years, and this was comparable in a teenage population [78,79]. Similarly, Villafaina et al. [80] examined 297 adolescents aged 11 to 12 years and found that girls had considerably lower levels of LS. However, a recent meta-analysis in children and adolescents reported that while boys had higher LS than girls, most studies found no significant differences [76], implying that girls perceive higher levels of LS in areas involving greater social interaction (school, family, and friends), while boys show better perceptions in comfort, social acceptance, self-perception, and self-esteem, according to Breslin et al. [77]. This might be conditioned by the multiple domains of LS measured, since male and female children and adolescents may have distinct views of certain domains of LS [76].

In terms of disparities in LS levels among young people and adolescents based on BMI levels, our findings are consistent with those published in the scholarly literature. Children who are overweight or obese have lower levels of LS. In this regard, Iturra and Sarrias [81] concluded in their study of 4518 children aged 9 to 12 years old that children with higher BMI, weight, and obesity levels had a negative relationship with LS, implying that the higher the body weight, the lower the satisfaction with life during childhood. According to Forste and Moore [82], higher levels of obesity impair the capacity for adolescents to self-evaluate their LS, with girls having lower levels of LS, with one of the main reasons being a pressure on women to be thinner, adding to the negative relationship between BMI and LS. As a result, according to Rosa Guillamón et al. [83], obesity may be a deciding factor in achieving a lower degree of LS. As a result, LS monitoring during school age is especially important since it declines with adolescence [84], and the influence of gender increases with age [85]. As a result, school age may be a critical period in mitigating such consequences.

Furthermore, multiple research works have demonstrated a link between LS and various domains [34,35,83,86], which is consistent with the findings which revealed a positive association between LS and each of the PL domains. Daily physical activity and LS have been linked in several groups [34,35], particularly in children and young people [36]. Similarly, in the domain of physical competence, Rosa Guillamón et al. [83] discovered that children with higher levels of physical fitness and physical exercise practice had higher levels of LS [87,88,89]. However, no research on the influence of PL on LS levels in children has been conducted. PL was favourably linked with major dimensions of emotional and social well-being in the adolescent population [44], and in undergraduates [47], researchers discovered that PL had a mediating influence on LS, which is consistent with the findings of Ma, Liu, Raymond Sum, Gao, Li, Choi, Huang and Xiang [48], who discovered a direct association between PL and mental health and resilience. As a result, and consistent with prior research and the findings of this study, given the direct association between PL and several components of LS, LS may play a role in PL.

Given that LS levels in children and adolescents have decreased in recent years (especially in girls and becoming more relevant with increasing age) [45], and given the direct relationship obtained with PL, the development of PL intervention programmes in children could be a beneficial measure in order to slow down the decrease in LS of schoolchildren and adolescents. Furthermore, because females score poorly in terms of both LS and PL, treatments that increase PL may be especially significant for girls.

One of the strengths of this study is that it was the first to show a direct relationship between PL and LS in children, although the latter has been related to several domains that compose the PL [34,35,83,86]. Therefore, it is a pioneering study in Spain on the relationship between LP and LF in children, as no previous studies have analysed these variables together. In addition, it uses the CAPL-2 to measure the level of participants’ PL, allowing it to be assessed comprehensively and objectively from its different domain. Finally, it evaluated the influence of sex and weight status on the variables studied, providing relevant information for designing strategies adapted to more vulnerable groups. Thus, this study’s findings have important implications for physical education, health promotion, and education policies. The integration of PL in school programmes, and its promotion and encouragement outside of them, could be crucial for participation in physical activities, promoting PA and achieving better health outcomes for children.

Because this is a convenience sample, one of the key limitations of this study is that the results cannot be extended to other groups. Furthermore, because the sample is from a specific location, its socioeconomic and cultural features may differ from those of other parts of the country. Another drawback is that causal links cannot be proven because of the typology of this cross-sectional investigation. Furthermore, owing to the inconsistency in gender differences in life happiness, utilising a global questionnaire does not specify the many characteristics that LS may have. The last limitation found in our study is the self-report questionnaire, which does not allow us to establish causal relationships, preventing us from establishing the objective level of the participant. As a result, the authors recommend that future studies employ a longitudinal design in order to establish such causal relationships, as well as to track the evolution of PL and its dimensions, as well as their relationship across various educational or developmental stages.

## 5. Conclusions

The current study showed that males had better PL and life happiness than girls, and that normal-weight children had higher LS than overweight and obese children. Furthermore, PL is related to a lower BMI and a higher level of LS. Except for the knowledge and understanding domain, all domains were related with LS. Furthermore, the daily activity domain is linked to gender, and the physical competence domain is linked to BMI. As a result, it is possible to conclude that gender, BMI, and LS are factors in the development of PL. Therefore, this article could serve as evidence for future interventions aimed at promoting physical activity and launching exercise programmes specifically for girls. This could help increase their levels of PL and overall happiness in life.

## Figures and Tables

**Table 1:** Physical literacy and life satisfaction scores segmented by sex and bodyweight group.

	All (*n* = 133)		Male (*n* = 62)		Female (*n* = 71)			Normal Weight (*n* = 74)		Overweight or Obesity (*n* = 58)		
	M	SD	M	SD	M	SD	*p*	M	SD	M	SD	*p*
Age	10.09	0.76	10.16	0.71	10.03	0.81	0.283	10.03	0.79	10.17	0.73	0.183
BMI	20.00	10.27	20.98	14.58	19.15	3.72	0.696	16.61	1.49	24.27	14.38	<0.001
Daily Physical Activity Behaviour (DB)	17.30	7.51	20.98	6.94	14.08	6.46	<0.001	18.05	7.37	16.33	7.64	0.208
Physical Competence (PC)	15.80	5.67	17.21	6.03	14.56	5.06	0.012	17.48	5.78	13.61	4.74	<0.001
Motivation and Confidence (MC)	25.57	2.98	26.25	2.64	24.97	3.14	0.020	25.78	2.88	25.30	3.10	0.463
Knowledge and Understanding (KU)	6.44	1.69	6.47	1.82	6.42	1.58	0.853	6.52	1.66	6.34	1.73	0.692
CAPL-2	65.11	12.77	70.91	12.17	60.05	11.06	<0.001	67.84	12.43	61.58	12.43	0.010
In most ways my life is close to the way I would want it to be	4.02	1.03	4.31	0.81	3.77	1.14	0.004	4.19	0.95	3.81	1.10	0.026
The things in my life are excellent	4.45	0.76	4.57	0.64	4.35	0.83	0.129	4.57	0.70	4.31	0.80	0.040
I am happy with my life	4.37	0.89	4.59	0.72	4.18	0.98	0.009	4.41	0.83	4.33	0.96	0.745
So far I have gotten the important things I want in life	4.14	0.90	4.48	0.59	3.86	1.02	<0.001	4.26	0.91	4.00	0.88	0.042
If I could live my life over, I would have it the same way	3.95	1.25	4.24	1.18	3.69	1.26	0.002	4.13	1.17	3.71	1.32	0.043
SWLS	20.93	3.74	22.18	2.67	19.86	4.19	0.001	21.54	3.46	20.16	3.96	0.023

BMI: body mass index; SWLS: Satisfaction with Life Scale adapted for children, CAPL-2: Canadian Assessment of Physical Literacy.

**Table 2:** Association between physical literacy and its domains and life satisfaction, gender, and BMI.

	β (95% CI)	T Statistic	*p*-Value	R^2^
CAPL-2				
SWLS	0.412	5.362	<0.001	0.386
Sex	−0.301	−4.089	<0.001	
BMI	−0.176	−2.406	0.018	
*Daily Physical Activity Behaviour (DB)*				0.272
SWLS	0.299	3.789	<0.001	
Sex	−0.358	−4.551	<0.001	
*Physical Competence (PC)*				0.194
SWLS	0.377	4.577	<0.001	
BMI	−0.166	−2.021	0.045	
*Motivation and Confidence (MC)*				0.211
SWLS	5.975	0.466	<0.001	

BMI: body mass index; SWLS: Satisfaction with Life Scale adapted for children, CAPL-2: Canadian Assessment of Physical Literacy.

## Data Availability

Data are contained within the article and Supplementary Materials.

## Supplementary Materials

The following supporting information can be downloaded at: https://www.mdpi.com/article/10.3390/children11020181/s1, Questionnaire S1: CAPL-2. Canadian Assessment of Physical Literacy (Spanish version).
