# Supplementary material for: Role of Satisfaction with Life, Sex and Body Mass Index in Physical Literacy of Spanish Children
Source: Children (Basel). 2024 Feb 1;11(2):181. doi: 10.3390/children11020181 (PMC10886828; doi:10.3390/children11020181)
Supplement: Supplementary file 1 [file children-11-00181-s001.zip › children-2845852-supplementary.pdf]

CAPL-2. Canadian Assessment of Physical Literacy

Evaluación Canadiense de la Alfabetización Física-2

(Spanish Version).

¿Qué opinas sobre la actividad física?

Cuando te preguntamos sobre la actividad física, nos referimos a cuando te mantienes activo, jugando o haciendo ejercicio. La actividad física es cualquier actividad que haga que tu corazón lata más deprisa o te deje sin aliento algunas veces.

¿Por qué estamos haciéndote estas preguntas?

Queremos saber lo que los/las niños/as como tú piensan sobre la actividad física, el deporte y el ejercicio.

Por favor, recuerda:

- ¡No hay respuestas correctas o incorrectas! Solo queremos saber lo que piensas.
- Si no sabes una respuesta, por favor pon tu mejor opción.
- Tómate todo el tiempo que necesites, no hay límite.

¿Qué es más como yo?

Para cada pregunta tienes que leer dos frases y luego:

1. **Primero, rodea la frase que más sea como tú.**

2. **Después, pon una cruz en el cuadradito correcto dependiendo de si es MUCHO, POCO, NADA para ti.**

NO HAY RESPUESTAS CORRECTAS O INCORRECTAS, SIMPLEMENTE DINOS LO QUE PIENSAS QUE ES **MÁS COMO TÚ.**

Ejemplo 2:

○ **A algunos/as niños/as les gusta jugar con el ordenador**	**PERO**	○ **A otros/as niños/as no les gusta jugar con el ordenador**
□ MUY VERDADERO para mi □ POCO VERDADERO para mi		□ MUY VERDADERO para mi □ POCO VERDADERO para mi

Recuerda, en cada cajita necesitas rodear lo que más es como tú y después poner una cruz en “realmente” o “parcialmente” cierto. Tómate tu tiempo y haz todo el formulario con cuidado. Si tienes dudas, solo pregunta.

¡ASEGÚRATE DE RELLENAR CADA PÁGINA!

¿Qué es más como yo?

○ **A algunos/as niños/as no les gusta jugar a juegos que requieren actividad física**		**PERO**	○ **A otros/as niños/as les gusta mucho jugar a juegos que requieren actividad física**		
□ MUY VERDADERO para mi	□ POCO VERDADERO para mi		□ MUY VERDADERO para mi	□ POCO VERDADERO para mi	

○ **A algunos/as niños/as son buenos en los juegos activos**		**PERO**	○ **Otros/as niños/as encuentran muy duros los juegos activos.**	
□ MUY VERDADERO para mi	□ POCO VERDADERO para mi		□ MUY VERDADERO para mi	□ POCO VERDADERO para mi

○ **A algunos/as niños/as no encuentran mucha diversión en los deportes**		**PERO**	○ **Otros/as niños/as encuentran diversión en los deportes**	
□ MUY VERDADERO para mi	□ POCO VERDADERO para mi		□ MUY VERDADERO para mi	□ POCO VERDADERO para mi

○ **A algunos/as niños/as se les da bien el deporte**		**PERO**	○ **A algunos/as niños/as no se les da bien el deporte**	
□ MUY VERDADERO para mi	□ POCO VERDADERO para mi		□ MUY VERDADERO para mi	□ POCO VERDADERO para mi

○ **A algunos/as niños/as no les gusta practicar deporte**		**PERO**	○ **Otros/as niños/as realmente se divierten practicando deporte**	
□ MUY VERDADERO para mi	□ POCO VERDADERO para mi		□ MUY VERDADERO para mi	□ POCO VERDADERO para mi

○ **Algunos/as niños/as aprenden fácilmente los juegos de actividad física**		**PERO**	○ **Otros/as niños/as encuentran difícil aprender los juegos de actividad física**	
□ MUY VERDADERO para mi	□ POCO VERDADERO para mi		□ MUY VERDADERO para mi	□ POCO VERDADERO para mi

Gracias por decirnos qué niños son más como tú.

Solo tenemos algunas preguntas más. Por favor vuelve la hoja.

¿Por qué eres activo/a?

Los niños y las niñas pueden ser **activos/as** haciendo todo tipo de cosas:

- Ejercicio (andar o mantenerse en forma)
- Jugar al aire libre o hacer cosas activas (como jugar en el parque)
- Deportes (como fútbol, tenis, baile o natación)

Debajo tenemos algunas razones por las que deberías mantenerte activo/a.

Por favor lee cada frase y dinos <u>cómo de cierto es</u> para ti.

Soy activo porque…

	NADA	POCO	A VECES	BASTANTE	MUCHO
Ser activo es divertido	☐	☐	☐	☐	☐
Me divierto siendo activo	☐	☐	☐	☐	☐
Me gusta ser activo	☐	☐	☐	☐	☐

¿Cómo te sientes siendo activo/a?

La siguiente sección tiene algunas frases que describen cómo las chicas y los chicos se sienten SIENDO ACTIVOS y HACIENDO COSAS ACTIVAS (como jugar a juegos que requieren actividad física, jugar al aire libre o hacer deporte).

Por favor lee cada frase y dinos <u>cuánto se parecen a ti cada una de ellas</u>.

	NADA	POCO	A VECES	BASTANTE	MUCHO
Cuando se trata de jugar a juegos que implican actividad física, creo que soy bastante bueno/a	☐	☐	☐	☐	☐
Creo que lo hago bien en las actividades comparado con otros/as niños/as	☐	☐	☐	☐	☐
Cuando se trata de ser activo/a, tengo buenas habilidades	☐	☐	☐	☐	☐

1. ¿Cuántos minutos cada día deberíais hacer actividades física que haga que vuestro corazón lata más deprisa y os haga respirar más rápido, como caminar rápido o correr? Que tiempo deberías estar activo en el colegio y también cuando estás en casa o en tu barrio.

a) 20 minutos.

b) 30 minutos.

c) 60 minutos o 1 hora.

d) 120 minutos o 2 horas.

2. Hay muchos tipos de aptitudes físicas. Una se llama resistencia, o capacidad aeróbica, o acondicionamiento cardiorrespiratorio. El acondicionamiento cardiorrespiratorio significa:

a) Lo bien que los músculos pueden empujar, tirar o estirar.

b) Lo bien que el corazón puede bombear sangre y los pulmones pueden proporcionar oxígeno.

c) Tener un peso saludable para nuestra altura.

d) Nuestra habilidad para practicar los deportes que nos gustan.

3. La fuerza muscular o resistencia muscular significa:

a) Lo bien que los músculos pueden empujar, tirar o estirar.

b) Lo bien que el corazón puede bombear sangre y los pulmones pueden proporcionar oxígeno.

c) Tener un peso saludable para nuestra altura.

d) Nuestra habilidad para practicar deportes que nos gustan.

4. Si quisieras mejorar en una habilidad deportiva (como darle una patada a un balón o atraparlo), ¿Qué sería lo mejor que podrías hacer?

a) Leer un libro de cómo darle una patada y atrapar un balón.

b) Esperar a ser mayor.

c) Intentar ejercitar o ser más activo/a.

d) Ver un video, recibir una clase, o tener un entrenador para que te enseñe cómo darle una patada y atrapar un balón.

5. En esta historia sobre Ana hay palabras que faltan. Elige las palabras del cuadro para rellenar los huecos que faltan en la historia. Solo puedes usar una palabra para rellenar cada espacio en blanco en la historia. Hay más palabras que espacios en blanco, así que no utilizaremos todas las palabras.

	**Divertida**	**Estira**	**Resistencia**
**Pulso**	**Respiración**	**Flexibilidad**	**Bueno**
	**Fuerza**	**Malo**	**Deporte**

Ana intenta mantenerse activa todos los días. Correr diariamente es bueno para su corazón y sus pulmones. Ana cree que la actividad física es __________________ y también es _____________para ella. Cuando entrena con su equipo ella corre más para mejorar su _____________. Su equipo también hace ejercicios como flexiones y sentadillas que aumentan su ________________. Cuando se enfría, ________________ para mejorar su flexibilidad y disminuir la frecuencia cardíaca. Después de hacer ejercicio, ella comprueba su frecuencia cardíaca lo que también llamamos ______________ .

6. Durante la semana pasada (7 días), ¿Qué días te mantuviste activo/a durante un total de al menos 1 h o 60 minutos por día? Cuenta todo el tiempo que pasaste haciendo actividades que aumentaron tu frecuencia cardíaca o que te hicieron respirar profundamente.

He estado activo/a durante **1** **2** **3** **4** **5** **6** **7** días.

Cuéntanos sobre ti…

<u>Por favor rodea un número o una palabra para cada pregunta.</u>

¿En qué curso estas?

Si no está en la escuela hoy, marque con un círculo el grado en el que ingresará el próximo día que irá a la escuela.

PRIMARIA						ESO
1º	2º	3º	4º	5º	6º	1º

Eres: niño niña

¿Qué mes es tu cumpleaños?:

Enero Febrero Marzo Abril Mayo Junio Julio Agosto Septiembre Octubre Noviembre Diciembre

¿Cuántos años tienes?:

7 8 9 10 11 12 13

¡Gracias por responder nuestras preguntas!

☺
